# Estimating Density and Temperature Dependence of Juvenile Vital Rates Using a Hidden Markov Model

**DOI:** 10.3390/insects8020051

**Published:** 2017-05-15

**Authors:** Robert M. McElderry

**Affiliations:** 1 Department of Biology, University of Miami, Coral Gables, FL 33146, USA; rmcelderry@ucla.edu; 2 Fairchild Tropical Botanic Garden, Miami, FL 33156, USA

**Keywords:** *Anaea aidea*, caterpillar demography, multi-state mark–recapture, state-space model, stage-structured matrix

## Abstract

Organisms in the wild have cryptic life stages that are sensitive to changing environmental conditions and can be difficult to survey. In this study, I used mark-recapture methods to repeatedly survey *Anaea aidea* (Nymphalidae) caterpillars in nature, then modeled caterpillar demography as a hidden Markov process to assess if temporal variability in temperature and density influence the survival and growth of *A. aidea* over time. Individual encounter histories result from the joint likelihood of being alive and observed in a particular stage, and I have included hidden states by separating demography and observations into parallel and independent processes. I constructed a demographic matrix containing the probabilities of all possible fates for each stage, including hidden states, e.g., eggs and pupae. I observed both dead and live caterpillars with high probability. Peak caterpillar abundance attracted multiple predators, and survival of fifth instars declined as per capita predation rate increased through spring. A time lag between predator and prey abundance was likely the cause of improved fifth instar survival estimated at high density. Growth rates showed an increase with temperature, but the preferred model did not include temperature. This work illustrates how state-space models can include unobservable stages and hidden state processes to evaluate how environmental factors influence vital rates of cryptic life stages in the wild.

## 1. Introduction

Many organisms have life stages that are difficult to observe, and these cryptic life stages may provide critical links in the life cycles [1,2,3,4]. Cryptic stages, however, are often poorly incorporated in population dynamic studies. Compared with more apparent stages, it is not well known if cryptic life stages are more or less sensitive to environmental stressors such as climate change, resource limitation, and predation. It is well known that alterations in an organism’s stage-specific vital rates (i.e., survival, growth, and reproduction) can have dramatic effects on its spatial distribution [5], phenology [6], and abundance [7]. With an exponential increase in the listing of endangered species and loss of biodiversity globally [8], it is increasingly important that we evaluate the influence of environmental stressors on all life stages and population dynamics if our goal is to provide better forecasting of population persistence to improve management strategies.

Incorporating cryptic life stages into empirical demographic models is feasible and stems from a rich history of mark–recapture (MR) statistical procedures. At the core of these procedures is the evaluation of the joint likelihood of individuals surviving and being detected [9,10,11,12]. Multi-state mark-recapture (MSMR) models extended MR procedures to estimate stage-structured matrix models [13], which are popular tools in population biology to represent the life cycle of organisms with discrete life stages [14]. This link is important because a variety of analytical tools exist to extract demographic and life history properties from age or stage-structured matrices [14] and these matrices are useful for predicting population viability [15]. More recently, MSMR analysis and stage-structured population matrix models are being evaluated using a class of models called state-space models (SSM) [16,17], effectively unifying all MR procedures [18,19]. An SSM describes two parallel time series via two probability distributions, a state model and an observation model, and the likelihood is achieved by integrating the joint distribution of both distributions over all states [18,19]. Perhaps the most appealing aspect of SSMs is the ability to model hidden states and hidden processes, e.g., Gimenez et al. [20] included uncertainty in breeding status as a hidden state. For the remainder of this paper, I focus on SSMs with discrete spaces; these are commonly known as hidden Markov models (HMM) [20,21].

An HMM is an ideal tool to analyze MSMR data for organisms with stages that are hard to observe in the wild [22,23,24]. A prime example of organisms with complex life cycles and cryptic life stages are Lepidoptera (butterflies and moths). Like many organisms, Lepidoptera undergo a series of discrete developmental stages. Newborn caterpillars hatch from eggs, develop through five instars, and pupate before emerging as winged adults. A majority of the demographic studies of butterflies focus exclusively on the adult life stage, presumably because juvenile stages are thought to be too difficult or delicate to study. Caterpillars for many species may be difficult to find or handle because they are small and cryptic, and for species with larger, more apparent caterpillars, mobility or molting may inhibit re-identification of marked individuals. Successful recruitment to the adult butterfly stage, however, is determined by juvenile survival and growth, which are sensitive to environment conditions, and juvenile stages for many species could be easier to study in the field than we think. The general lack of a complete life cycle in modeling butterfly population dynamics motivates this study and displays how HMMs help circumvent problems of missing and/or imperfectly detected states when applying MSMR methods to caterpillar monitoring in the field. Specifically, by manipulating the observation model of a HMM, we can account for differing observation probabilities among stages. More apparent stages will have high probabilities of being observed, which decrease to smaller probabilities and even zero for completely hidden stages. Meanwhile, the demographic model that parallels the observation model in the HMM maintains all stages whether or not they are observed.

It is particularly important that we identify environmental factors that influence juvenile dynamics to understand the response of butterflies to climate change [4] and for prioritizing restoration actions and cultivating persistence of rare and endangered butterflies on the brink of extinction [25,26]. However, few studies have included juvenile vital rates to evaluate rare insect population dynamics (however, see [27,28]). The most commonly used method to assess how environmental factors influence the population dynamics of rare insects is to correlate environmental variables with the number of adult insects observed (e.g., [25,29,30,31]). This method searches for simple correlations between variables that result from multiple processes with complex interactions and feedback. However useful this method has been in rapid assessments and mining existing data sets, resulting models tend to lack statistical power and suffer from overfitting [32]. A mechanistic approach, in which specific vital rates are linked to environmental variables, appears a more promising means for evaluating environmental drivers of population dynamics (e.g., [4,33]).

In this study I used HMM and Bayesian procedures to quantify the survival and development of *Anaea aidea* caterpillars from egg to pupation. Additionally, I quantified how underpinning environmental drivers influenced the temporal variation in these vital rates. *Anaea aidea* (Lepidoptera; Nymphalidae) was selected for this study, in part, to help develop a restoration plan for an endangered butterfly, *Anaea troglodyta floridalis*, which is phylogenetically and ecologically similar to *A. aidea* and is currently too rare to be studied directly [34]. *Anaea t. floridalis* is endemic to the endangered pine rockland ecosystem of southern Florida where juvenile mortality of *A. t. floridalis* exceeds 70% [35]. In concert with other environmental stressors, such as atypical frosts and alterations to the timing and frequency of wild and prescribed fire, high juvenile mortality may be a primary driver of *A. t. floridalis* population decline [31]. However, little data exists describing *A. t. floridalis* life history and sensitivities of vital rates to disturbance and the environment. To fill in this data gap, I monitored a complete recruitment pulse of *A. aidea* during spring from the appearance of eggs to the disappearance of caterpillars in search of pupation sites. To represent *Anaea*’s life cycle, I developed a periodic stage-structured matrix model of population dynamics that projects the population through multiple generations over one year in 3-day time steps (see [36]), and I describe here how I estimated the juvenile vital rates for this periodic model while testing each vital rate for the effects of temperature and conspecific density.

## 2. Materials and Methods

### 2.1. Study Species and Field Methods

*Anaea aidea* (tropical leafwing) is distributed throughout Central America and Mexico in tropical dry forest [37], and a population has persisted many years in Austin, TX, USA, (personal communication [38]). I surveyed *A. aidea* on its local larval host plant, *Croton fruticulosus*, in 14 randomly selected (see Appendix A) host-containing plots at the University of Texas Brackenridge Field Laboratory (BFL), which comprises 82 acres along the Colorado River in Austin (30${}^{\circ }$17${}^{\prime }$00${}^{\prime \prime }$ N, 97${}^{\circ }$46${}^{\prime }$44${}^{\prime \prime }$ W). Suitable habitat at the BFL was similar in appearance to habitats for *A. aidea* and *C. fruticulosus* observed in larger, less-disturbed natural areas west of Austin, i.e., oak-cedar woodland on limestone outcrop upslope from riparian hardwood forest and meadows on alluvial terraces. In each plot I searched each tagged *C. fruticulosus* shrub for *A. aidea* caterpillars at each of nine survey dates. Three days were required to survey all plots, so I resurveyed each plot every three days beginning 28 March 2011 and continued every day until 23 April 2011. All *A. aidea* caterpillars encountered were included in the survey, and I tracked each individual caterpillar through time recording its developmental stage (egg, one of five instars, or pupa), length, and status (live or dead) at each survey date. Mark–recapture of caterpillars is rare (however, see [29,39]) since caterpillars shed their skins between developmental stages. Although egg and pupa stages were largely unobservable, the particular behavior of *A. aidea* caterpillars provided an opportunity for marking the leaf where they were found, so they could be relocated in subsequent surveys (see [27] and Appendix A). Typical of MR analyses, I arranged the string of observations for each caterpillar over all dates as an encounter history.

### 2.2. Model Description

I represented all possible states (live, newly dead, dead) and stage (egg, first to fifth instar, pupa) in a column stochastic model ($P_{X}$) that describes all possible transitions among states and stages [16,20]. In a column stochastic model, all columns sum to one, indicating that all possible fates are included [14].

(1) $$P_{X}=\left[\begin{array}{cc} J & Z\\ M & U \end{array}\right]$$

The matrix, $P_{X}$, is a block matrix consisting of matrices J, M, Z, and U, and contains the transition rules for a Markov process whereby an individual’s current state and stage predict its future state and stage.

**Figure 1:**
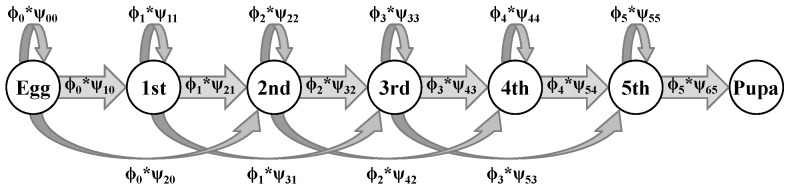
Diagram of the juvenile portion of the leafwing life cycle (*Anaea* spp.) with circles indicating each developmental stage, and arrows indicating the one-time step probabilities of transitioning ($\psi_{ji}$) from stage *i* to stage *j* conditional on survival ($\phi_{i}$).

Matrix J represents probabilities of growth among juvenile stages that remain alive. Each element is a product of survival and growth and corresponds to an arrow in the life cycle graph for *A. aidea* juveniles (Figure 1). In 3-day time steps, there is a stage-specific probability of surviving, $\phi_{i}$, and a stage-specific probability of growing from stage *i* to stage *j*, $\psi_{ji}$, independent of survival. Individuals can either remain in the same stage, j=i (called stasis), grow to the next stage, j=i+1, or early instars may grow two stages, j=i+2.

Matrix M represents all possible transitions from live stages to newly dead stages, and utilizes the same stage-specific survival and growth probabilities. Living individuals at time *t* die with probability $1-\phi_{i}$ and are partitioned into newly dead stage classes, e.g., newly dead first instar, etc. I distinguished between newly dead and dead, because newly dead individuals can be encountered in the field, but quickly disappear. Similarly, newly dead individuals transition to the dead state, which is absorbing and is represented by the last row in matrix M [40].

The remaining two matrices, Z and U, have appropriate dimensions and serve to enforce the absorbing state. All elements of Z are set to zero, which represents the impossibility of transitions from dead to live states. Similarly, all elements of U except for the last column are zero to prevent transitions among newly dead states. Elements in the last row of U are one and represent individuals in the newly dead state transitioning to the absorbing state.

While the block matrix $P_{X}$ describes the underlying state process of interest, matrix $P_{O}$ describes the parallel process by which I observe individuals. Matrix $P_{O}$ contains the probabilities of being observed ($p_{i}$) for all live and dead stages along the sub-diagonal. The columns of $P_{O}$ correspond to the true stage of an individual and the rows represent the possible stages in which an individual can be observed. The probability of not being observed is represented in the top row and is the complement of each stage-specific probability of being observed, i.e., $1-p_{i}$ (see Appendix B).

### 2.3. Vital Rates and Covariates

Survival in each stage *i*, $\phi_{i}$, was modeled either as a constant or as a linear function of survey date or caterpillar density (see Appendix C). I used survey date as an unspecified temporal effect, i.e., the simple progression of time, to represent the underlying myriad of changes occurring during spring as a linear progression. This was an attempt to capture all possible environmental changes together to contrast each specific component, caterpillar density and temperature, that changes during spring. I represented caterpillar density at the individual plant level either as the number of caterpillars or the sum of all caterpillar lengths to account for caterpillar biomass (see Appendix A). These density metrics correspond to two mechanisms by which density dependence may be realized. Numbers of caterpillars may be an attractor for foraging predators, or could provide safety in numbers, given a constant number of predators. Increasing caterpillar biomass per plant shows not just an increase in numbers but also of caterpillar size, and larger caterpillars consume more foliar biomass than smaller caterpillars.

Growth from stage *i* to stage *j*, $\psi_{ji}$, was modeled either as a constant, a function of survey date, or a function of local air temperature. Temperature is known to affect development time in insects, with increasing temperature generally increasing rates of metabolism and consumption resulting in quicker development [41]. To quantify the effect of temperature on development time of *A. aidea*, I obtained air temperature data collected every hour for the Austin area from the National Weather Service [42] and averaged hourly temperatures over three-day intervals corresponding to the survey intervals for each site. I used the logit link for all survival functions and the multinomial logit for growth to allow for transitions to multiple stages while requiring that the probabilities of all possible transitions from *i* to any stage *j* sum to one (Appendix C).

### 2.4. Model Likelihood

Two equations comprise the likelihood of this hidden process model. The state equation describes the state of an individual at time *t* given its state at time t−1 and $P_{X}$. The observation equation describes the probability of being observed at time *t* given the state of an individual at time *t* and $P_{O}$.

(2) $$\begin{array}{cc} X_{k,t}|X_{k,t-1} & ∼Categorical(P_{X}X_{k,t-1})\\ Y_{k,t}|X_{k,t} & ∼Categorical(P_{O}X_{k,t}) \end{array}$$

Each individual *k* at each time t−1 was represented by a column vector $X_{k,t-1}$ of zeros with a one in the row that indicates an individual’s state. The state of an individual at time *t* was predicted by the vector of probabilities obtained by multiplying $P_{X}$ by $X_{k,t-1}$. Similarly, the observed state of individual *k* was contained in matrix $Y_{k,t}$, and the probability of being observed in each state given its actual state is obtained by multiplying $P_{O}$ by $X_{k,t}$. The categorical distribution is a special case of the multinomial distribution for a single sample and is appropriate here since these equations were evaluated for each individual at each time step.

I used Bayesian estimation via Markov chain Monte Carlo (MCMC) simulations to fit the model above to encounter histories of marked caterpillars surveyed in the field. For linear model parameters, e.g., $b_{1i}$, $a_{1i}$, etc. describing survival and growth probabilities, I used uninformative normal prior distributions with mean zero and standard deviation equal to 100. I also specified uninformative priors for observation probabilities as uniform between zero and one. Demographic parameters for eggs and pupae, including the fifth instar to pupa transition, were rarely observed but necessary for model stability, so I estimated informative prior distributions using data from lab-reared larvae (see Appendix D). The posterior distributions for all parameters were estimated using 10,000 MCMC iterations without thinning following 120,000 iterations of ‘burn in’, which according to standard convergence diagnostics was sufficient for model convergence. I verified model convergence using visual checks of posterior distributions, and by calculating the Gelman–Rubin potential scale reduction factor (psrf) for each parameter and a multivariate psrf, all of which were close to one. I additionally calculated the multivariate expected sample size (using the R package mcmcse), which for the top two ranking models were 4204 and 3587. I performed all procedures using a high-performance computing cluster (533 h) and the programs R (version 3.1.0 (10 April 2014) [43]) and JAGS [44], controlling JAGS via the R package, rjags. It should be noted that other methods have been proposed to fit HMMs using maximum likelihood calculations [23], which could greatly reduce computation time compared with the MCMC methods presented here. However, MCMC methods provide a simpler means for credible interval estimation compared with profile likelihood or bootstrapping methods to estimate confidence intervals for maximum likelihood estimates.

Constructing 18 competing models I used Akaike’s information criteria corrected for small sample size (AICc) to evaluate the relative support of candidate models [45]. I selected to use AIC over DIC (deviance information criteria), because the estimated number of effective parameters was negative for some models, a known weakness of DIC [46]. Gosky and Ghosh [46] suggest AIC over DIC and used the mean posterior deviance in place of the deviance from the likelihood that is typically used in calculating AIC. With the competing models, I investigated the effects of date and density on survival and the effects of date and temperature on growth. I also tested for reduced stage structure in survival, grouping first to fourth instars as distinct from fifth instars. In all models, the probability of observation was stage-dependent and constant. It is important to note that I evaluated the relative fit of each complete demographic model rather than evaluating each vital rate–environment relationship independently. Each candidate demographic model contained a unique combination of vital rate–environment relationships, representing a competing hypothesis of environmental sensitivity integrated within the system’s dynamics. With this approach, the relationship between each vital rate and the environment was evaluated in the context of all other vital rates.

## 3. Results

**Figure 2:**
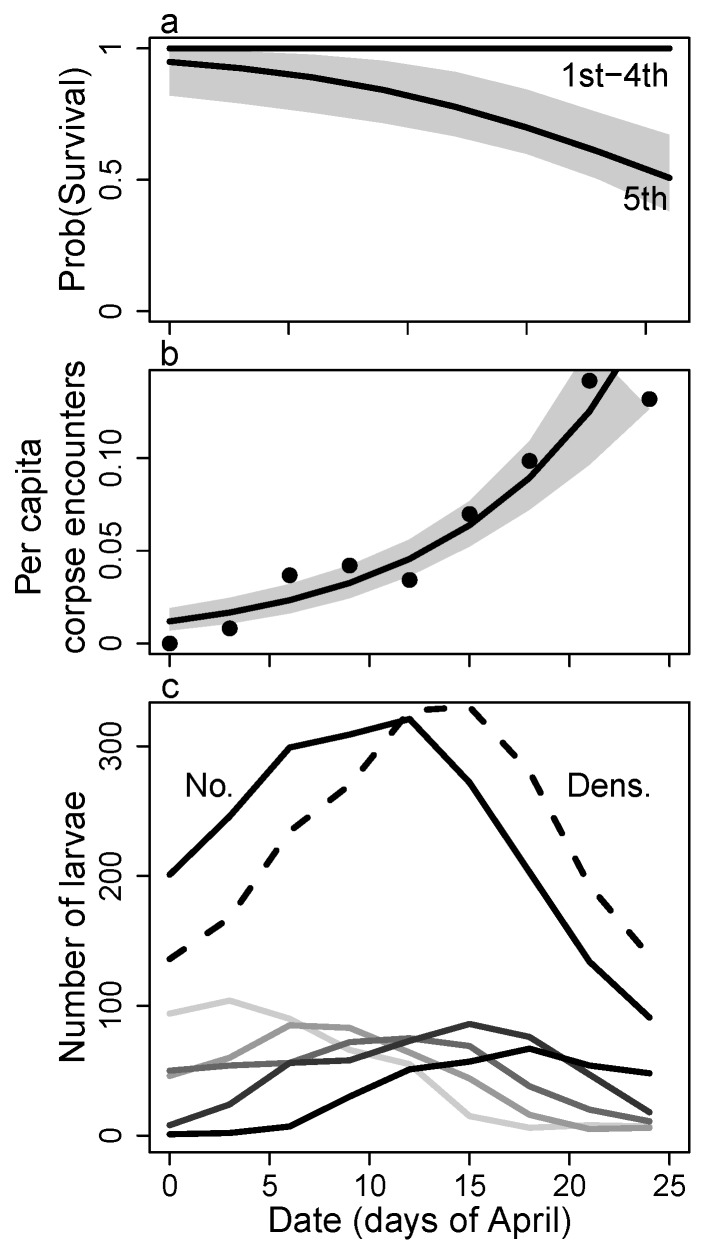
Change in (**a**) the mean probability of survival; (**b**) the per capita corpse encounter rate, and (**c**) the number of larvae in each stage and total caterpillar biomass for *Anaea aidea* over the study period. Gray shaded areas represent the 95% CI. Points in (**b**) represent the number of caterpillar corpses encountered per capita in each survey, and the line represents the encounter rate; fit using Poisson regression. Upper lines in (**c**) show the total number of caterpillars (No.) and the size-scaled density (Dens.) over time, while the lower lines show the number of caterpillars in each instar. From light to dark, these lines represent early to late instars.

**Figure 3:**
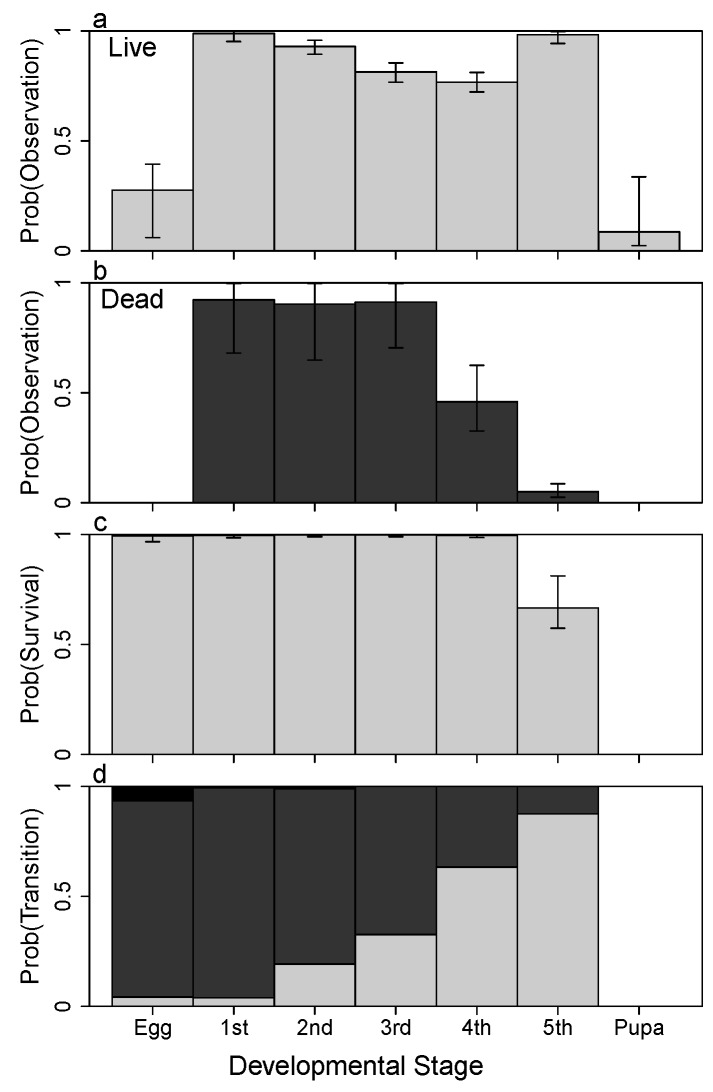
Stage-specific mean probabilities of (**a**) detection for live (light bars) and (**b**) dead (dark) individuals; (**c**) survival; and (**d**) transitioning from each stage *i* to either *i* (light), i+1 (medium), or i+2 (dark) during a 3-day interval for *A. aidea*, independent of survival. Error bars indicate the 95% credible interval.

Caterpillar abundance increased during this study to a maximum density in mid-April, and then decreased as new individuals appeared less often and either died, left the host plant to pupate, or left the host plant and the study area (Figure 2c). Over 27 survey dates (9 surveys plot${}^{-1}$), I observed 510 individuals in a total of 2183 observations. I observed both live (n=2062 observations) and dead (n=107) caterpillars with relatively high probability (Figure 3a), but I seldom observed eggs and pupae (n=14). The probabilities of observing live individuals varied by stage, with third and fourth instars observed with a lower probability compared with first, second, and fifth instars, which may be due to the shift in perch construction behavior from frass chain to leaf roll during these intermediate instars. The probabilities of observing dead individuals were relatively high, but these probabilities dropped significantly for fourth instars and more so for fifth instars (Figure 3a). Caterpillar corpses encountered most commonly showed signs of predation, i.e., a blackened and somewhat shriveled body at a single feeding point, but there were a few corpses found that died during the molting process. Failed molting was indicated by a persistent head capsule from the previous instar stuck over the mouthparts of the new instar head capsule. In these instances, the caterpillar successfully survived the previous stage and starved in the new. I recorded these as deaths in the new stage.

Survival of fifth instar larvae decreased over time, and AICc favored a simple model (Table S1) with early instars sharing one constant survival rate (Figure 2a and Figure S2b). Survival started high for all stages, but steadily declined through the growing season for fifth instar larvae (Figure 2a). Survival appeared to be linked to density, but survival increased rather than decreased with high density (Figure S1). Models that used the size-scaled density (the summed length of caterpillars per plant) outperformed models that used the number of caterpillars per plant. For simplicity, only models with size-scaled density were used for model evaluation (Table S1). In general, density models did not receive significant support relative to other models. Mean caterpillar length increased almost 5-fold during larval development from first to fifth instars (first = 4.1 mm, second = 6.4 mm, third = 9.4 mm, fourth = 13.3 mm, fifth = 19.9 mm). Total size-scaled density lagged a few days behind the total number of caterpillars as each of these metrics increased to a maximum midway through the brood then decreased (Figure 2c). Over this time period, stage structure progressed from early instar predominance to late instar predominance.

Per capita predation rate, measured by Poisson regression of the number of corpses encountered over time, increased by 11% per day during the month of April (see Appendix D). Thus, individuals late in the brood faced higher risk of predation than individuals early in the brood (Figure 2b). Most dead caterpillars looked like they had been attacked by one of several insect or arachnid predators (i.e., the corpses were left clinging to the plant and exhibited evidence of feeding damage). I observed numerous hemolymph-sucking arachnid and hemipteran predators patrolling for caterpillars. Hemipteran predators included a wheel bug (Reduviidae) and a soldier bug (Pentatomidae), both of which hatched from eggs laid on *Croton* and appeared to focus on attacking *Anaea* caterpillars during each bug’s larval development. Additional insects patrolling *Croton* and/or attacking *Anaea* caterpillars include green lacewings (Chrysopidae) and a small parasitoid wasp (Hymenoptera). Large polistine wasps (Polistinae) were seen searching *Croton*, and I observed several yellow jackets (Vespinae) attacking a large caterpillar of another species, but I did not observe predation events of *Anaea* caterpillars with these potential predators. Not observing predation by large predators such as wasps and birds does not mean that these predators did not have an effect. These predators tend to remove caterpillars from where they are found, leaving little if any trace (personal observation [47]). There was a single instance where a rolled leaf shelter appeared chewed by a beak, leaving triangular markings and black hemolymph as the only remains. Predation rates by wasps and birds could not be quantified in this study. Disappearance of fifth instar larvae could mean either predation or pupation, but I controlled for this in the modeling by using an informative prior distribution for the probability of pupating (see Appendix C).

**Figure 4:**
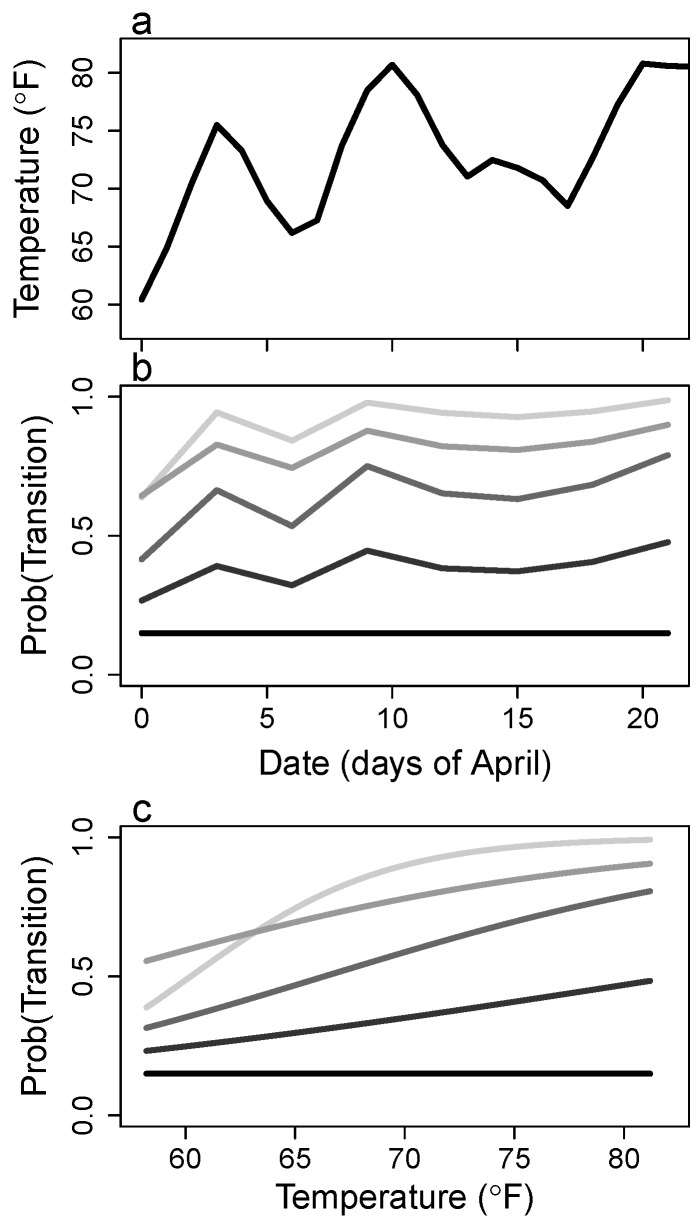
Change in (**a**) mean air temperature and (**b**) the probability of growing during a 3-day interval for *A. aidea* over the study period. The change in growth probabilities over time (**b**); is due to the relationship between transition probabilities and temperature (**c**). Temperature represents the hourly regional air temperature averaged over each 3-day time step. From light to dark, the lines in (**b**,**c**) represent early to late instars.

The probability of growth (advancing either one or two stages) within 3 days decreased for progressively higher stages (Figure 3c and Figure S2c). Concomitantly, the probability of remaining in the same stage for 3 days increases for later instars. Data for fifth instars pupating were insufficient to differentiate the posterior estimate from the prior distribution, so this description excludes fifth instars. Over the month of April, the probabilities of growth generally increased, mostly for early instars (Figure 4b). This increase in growth rates represents more rapid development and was linked to increasing temperature (Figure 4). Temperatures showed a warming trend during this survey, increasing on average about 20 ${}^{\circ }$F, but there was also significant temperature variation over time (Figure 4a). Warmer temperatures resulted in increased growth for all stages (Figure 4c). Despite a clear relationship between temperature and growth rates, model selection results suggested that neither temperature nor the progression of time in April were significant predictors compared with models that included only stage-dependent growth rates.

## 4. Discussion

Evaluating the sensitivity of cryptic life stages to environmental stressors is a major challenge as we develop conservation plans that account for climate change. Although we are often not able to monitor cryptic stages directly, SSMs easily incorporate hidden states and processes within demographic models and facilitate the evaluation of how changing environmental factors influence all life stages. My work displays the utility of SSMs in dealing both with imperfect detection and rarely observed stages and transitions in a butterfly system. Both eggs and pupae stages were largely unobserved, making estimation of their demographic rates problematic without non-cryptic stages preceding and following these cryptic stages. By adding laboratory-derived estimates for the probability of pupation, I was able to tease apart the probabilities of a fifth instar dying or pupating, both of which result in disappearance from the study. However, I was unable to obtain estimates of pupa survival and maturation rates without adult recruitment observations. In addition to providing an example of how SSMs can be used to obtain a more precise representation of butterfly population dynamics, this work contributes to a growing body of literature focused on revealing the demographics of cryptic life stages. SSMs have been employed to reveal animal movement patterns [21,48], the importance of dormancy in plants [49], and uncertainty in either breeding [20,22] or infection status [50]. SSMs are flexible, making them especially useful for ecological modeling. For example, Schaub and Abadi [51], have developed a unified demographic modeling framework, called the integrated population model, that uses an SSM to integrate time series count data with focused demographic data. Integrated population models will be particularly useful for insects to integrate field counts of a single life stage with laboratory data for all other stages to obtain a complete empirical model of the life cycle and to evaluate within- and between-year trends. Integration of available data while considering each life stage and accounting for all sources of uncertainty is becoming increasingly important to understand how species will respond to future environmental conditions, especially for ectotherms (see [4]).

In this study, I applied an HMM using Bayesian procedures to estimate vital rates among juvenile stages from field surveys of caterpillars and developed a stage-structured demographic matrix that describes the butterfly recruitment pulse, which is not well quantified in most butterfly demographic models [31,52]. Although maximum likelihood procedures may expedite fitting HMMs [23] compared with computationally expensive MCMC methods, confidence limits are more efficiently obtained from MCMC. Furthermore, with a full life cycle model, the effects of parameter uncertainty on the population growth rate, the dominant eigenvalue of the transition matrix, are easily evaluated using MCMC [49]. Here, I used the state-space framework to model error in both observation and state processes while including individual level covariates and data from multiple sources [18,19,53]. Variation in each vital rate was modeled via stochastic simulation (MCMC) for each individual at each survey (demographic stochasticity), independent of variation in observation probabilities and whether or not it was observed (observation error). Separating sources of error improved precision of demographic estimates and revealed patterns in the vital rates due to caterpillar density, temperature, and the progression of time over the month of April.

Although survival appeared to increase at high caterpillar density, and growth rates increased at high temperatures, models including these relationships were ranked poorly by AICc. Using this approach, the likelihood of all growth and survival probabilities for all stages and covariates were evaluated simultaneously, such that the effects of each vital rate-covariate relationship were tested within the context of the entire model. What this means is that these results say less about whether survival or growth were affected by density or temperature, and more about which covariates were important in describing the dynamics of this system given the current model set and data. Model selection using AICc suggested that the recruitment process for *A. aidea* was best represented either with stage-specific survival and growth rates constant over the month of April or with constant survival for early instars and decreasing survival for late instars over the month of April. This result does not suggest that density and temperature had no effect on the survival and growth rates of caterpillars but does indicate a simpler model characterized variation in the observed data more efficiently [45]. Although a stage-structured model of a Markovian process provides a simple and convenient tool to describe the discrete life cycle of butterflies, the inherent discretization of a continuous process, such as the time to each stage transition, may not be the best approach if the purpose of this research had been to explore temperature-dependence in growth rates (see discussion of growth below).

### 4.1. Detection

Overall high observation probabilities for all living caterpillar stages and caterpillar corpses lend credibility to estimates of vital rates reported here. High observation probabilities estimated for caterpillar stages reflect the high fidelity of caterpillars to a particular area on a host plant, which was likely due to the time invested in building perches [27]. First to third instar larvae constructed frass chain perches extending from the leaf tip and generally remained on a single leaf for each stage. Third instars typically molted on the frass chain and then moved to a new leaf to construct a leaf roll as a fourth instar. This shift in perch types may explain why I observed third and fourth instars less frequently. Fourth and fifth instars rolled leaf shelters and fed in an increasing area around this shelter. Misidentification was rare, and when I could not correctly distinguish between individuals, I treated them as new individuals (see Appendix A). As caterpillars grow progressively larger, they require more foliar biomass to complete development in each stage. In the laboratory, third, fourth, and fifth instars required approximately 2, 5, and 20 average-sized leaves, respectively, to complete development (unpublished data [54]). Motivated by increasing nutritional requirements, later life stages became progressively more mobile in the field, and fifth instars often defoliated all or a large portion of leaves on their host plant and then moved to a new perch or host plant. Toward the end of the survey, host plants were increasingly defoliated and hungry caterpillars either moved to adjacent host plants or were not observed again.

Typical MR estimates represent apparent survival, which is the joint probability of surviving and remaining in the survey area [12]. From the abundance of observed dead first to fourth instars I was able to estimate true survival (see [55]) for these stages, but I found substantially fewer fifth instar corpses. This was complicated further by the fact that all surviving fifth instars eventually leave the host plant to pupate. Dead fifth instars and pupa are therefore hidden states, and I used prior information to bolster estimates of pupation probability in order to estimate true survival for fifth instars. Specifically, I supplied an informative prior distribution for pupation probability estimated from individuals in the laboratory (see Appendix C).

Incorporation of prior information to bolster estimates for hidden states underscores the benefit of using a Bayesian approach. In cases such as this with limiting data, demographic parameter estimates may not be identifiable [56] using field data alone, but laboratory rearing may provide reliable estimates that are easily specified as prior distributions. In my study, egg development rate and probability of pupating were unidentifiable parameters due to the rarity of observing these hidden stages. I did not allocate time to searching for eggs, often hidden on the undersides of leaves, nor did I search for dead fifth instars or pupae on neighboring plants and leaf litter. Here, I incorporated data from lab-reared caterpillars, which is an efficient strategy for many insects and other short lived organisms. Doing so bolstered unidentifiable parameters, thus improving model stability and identifiability of confounded parameters, e.g., apparent survival.

### 4.2. Survival

Fifth instar survival declined over the study, suggesting a benefit to being born earlier. This brood was surveyed from the end of March to the end of April, a time during which overwintering adult *Anaea* become active and several changes occur while spring progresses in Central Texas. The temporal effect I included in the model represents this spring progression. Warming temperatures is a primary driver of several changes at this time, e.g., bud break, flowering, and the emergence of insects. In this study, it is likely that a subset of environmental factors, such as an increase in insect abundance [57] and/or increased nesting and breeding of predatory birds [58], was the driver of decreased survival of caterpillars during the month of April. Although models that included density as a covariate received significantly less support than the time-dependent survival model, the complex relationship between density and survival may also help explain the mechanisms underlying the temporal decline in survival. Oviposition rate decreased and predation rate increased as the brood progressed. This trend may explain the observed initial increase in caterpillar density followed by the slow decrease over time. There are two ways density could have affected survival in this system (see [59]). Caterpillars increased in size and numbers, which decreased the amount of leaf tissue available for consumption. Similarly, predator numbers and sizes seemed to increase in response to high caterpillar density, which could have resulted in an increase in the per capita predation rate.

The estimated effect of density on survival was interesting in that it implied that caterpillars were safer in high density, but unlike caterpillars that gain increased growth rates and reduced predation by feeding in aggregations (e.g., [60]), *Anaea* caterpillars are solitary. Rather than caterpillars benefiting from safety in numbers, this result was better interpreted in terms of predator–prey dynamics with predator dilution or satiation at high prey density. In classic models of predator–prey dynamics, predator numbers lag behind prey numbers and predation rate increases with the rise in predator density [61]. Increasing numbers of predators coupled with decreasing prey yields an increasing per capita death rate for the prey, which is what I found. The *Anaea* recruitment pulse was followed by a predator recruitment pulse, meaning that *A. aidea* caterpillars were threatened by fewer predators early in the brood, and predation pressure increased as the predator density increased over time.

Despite support for predator–prey dynamics and positive density dependence, the alternative hypothesis of resource competition among prey for limiting resources should not be ruled out. I was unable to include the lasting effect of previous caterpillars in the metric of density employed here, which would be crucial to evaluate the influence of limiting resources and negative density dependence. While caterpillar density increased then decreased during the survey, none of the leaves consumed by caterpillars were replenished, meaning the abundance of edible leaves simply decreased over time. The abundance of leaves was not measured, but this resource certainly decreased in the month of April, possibly contributing to the observed decline in survival over time. However, only small shrubs were completely defoliated. In these relatively few instances, adjacent shrubs with foliage were present nearby. Finding starved caterpillars would have supported this argument, but only caterpillars wandering away from available hosts would have starved, making them almost impossible to find. Fifth instars were the most likely to starve, given their much higher nutritional needs relative to early instars, and were also the least likely to be observed dead. In a study of the gypsy moth that is known for dispersing as caterpillars, Weseloh [39] showed that caterpillars dispersed in random directions, and late instars traveled no further than 15 m between captures. In my study, successful dispersal between patches seems unlikely given that only one or two directions lead from one host patch to another, and adjacent patches were at least 50 m apart. Furthermore, I have never observed caterpillars off of their host during several years of repeated surveys at this site. With the multiple complexities described here that may affect density dependence, it is not surprising that caterpillar density was not supported in the preferred models. Caterpillar, predator, and leaf density are all likely to interact, and time was the only variable that seemed to effectively capture the change in these variables. Support for a temporal decline in survival over an effect of caterpillar density does not refute density dependence, rather it suggests that density dependence may be more complex than the simple linear function of density employed here.

The preeminence of predation as a cause of death for *A. aidea* juveniles is interesting not only for *A. aidea*, but it has implications for *A. t. floridalis*, for which a relatively large number of predators and parasitoids have been described [35]. What is interesting is that *A. t. floridalis* is the only leafwing that does not construct rolled leaf shelters as fourth and fifth instars. All other *Anaea* (and many closely related genera) construct shelters either by rolling single leaves or tying leaves together with silk, presumably to evade parasitism and predation [37]. *Anaea t. floridalis* larvae have been observed tying leaves together, but this behavior is observed very rarely [62], and may be due in part to the host plant architecture. The leaves of *Croton cascarilla* are generally too slender to roll and too sparsely arranged to tie multiple leaves together into a shelter, which is not the case for the leaves of *Croton* used by other *Anaea*. Without the protection of a leaf shelter, late instar *A. t. floridalis* are more exposed to predators, and the predation rates measured for *A. aidea* in this study likely underestimate those for *A. t. floridalis*. Indeed, predation/parasitism was listed as the most common cause of *A. t. floridalis* larval mortality in the field [35].

### 4.3. Growth

Growth rates were stage-dependent and time-invariant according to AICc, which suggests development time was not extremely variable over the month of April. However, it is well known that ectotherm development is strongly affected by temperature [63], and there a few reasons why the model in its current configuration failed to capture temperature dependence. First, it is difficult to parsimoniously specify a mechanistic model describing development as a function of temperature (e.g., [63,64]). These models contain many more parameters than were included in the simple linear model presented here, and for suitable model fits, a range of temperatures including extremes is needed to fit non-linear thermal response curves for development, i.e., unimodal and skewed [63]. Additionally, estimating temperature effects is difficult in fluctuating field environments without adding some of sort of time lag. I opted for simplicity in the linear model form as a more conservative approach to avoid over parameterizing the HMM, and because a linear model may approximate the thermal response within the relatively small temperature range during this study. Exploring a more mechanistic approach to temperature dependence would be a major improvement in HMM modeling of insect population dynamics, but doing so would require more extensive field surveys and a more accurate representation of the development process.

Using a matrix model without specification of developmental delays may have weakened the power of this framework in detecting the effect of temperature. In the current framework the fate of an individual depends only on its current stage, and there is no memory of the stage duration for that individual. As caterpillars develop, they grow larger and increase feeding rates to meet increasing nutritional requirements for each successive molting [65].The time required in each stage therefore increases in later instars, represented here by increased probabilities of remaining in the same stage for later instars. Treating the continuous developmental process as a discrete process in a matrix model may have worked well for early stages where the stage duration roughly equals the survey time interval, but likely did not work well for later instars. This point may explain the stronger response of first and second instars to temperature and weakened responses for third and fourth instars (Figure 4). I did not attempt to estimate temperature dependence for fifth instars, because I used laboratory data for this growth rate. The current framework could be improved by adding developmental delays explicitly in the model framework, e.g., [66], but current methods in time delay modeling would need to be adapted to deal with fluctuating environments.

## 5. Conclusions

This work contributes to the discussion of the drivers of population dynamics by analyzing density and temperature dependence within a comprehensive model of cohort dynamics while explicitly modeling individual stochasticity. Applying SSMs to analyze MSMR data is still relatively new and conveniently separates observation error from the demographic variation of interest. This level of detailed field observations and cohort model sophistication is more common in other animal systems (e.g., [16,23,50,51]), but is unprecedented in butterfly research and has proven effective in illuminating the progression of individuals from hatching to pupa. The recruitment process from birth to adulthood is lacking from most butterfly and insect field studies, but it is a crucial component when searching for links between ‘noisy’ time series of adult population counts and underlying demographic and climatic drivers. My work displays an SSM framework for estimating a stage-structured demographic matrix that includes hidden stages and the effect of climatic and demographic drivers from time series data with missing observations. However, my work also highlights how an oversimplified process model can weaken the ability to detect climatic effects. Future work could improve on this model by adding developmental time delays and more mechanistic thermal response functions. Still, the many features offered by SSMs that I highlight here make them particularly useful in studying life history and population dynamics in nature. This is especially true for insects, which are an exemplary example for organisms with low detectability and hidden states. Quantifying the effects of environmental factors on cryptic life stages and population dynamics will become increasingly important for accurately predicting how species will respond to environmental change.

## Supplementary Materials

The following are available online at http://www.mdpi.com/2075-4450/8/2/51/s1, Figure S1: Density-dependent survival results, Figure S2: Untransformed parameter values, Table S1: Model selection results, Supplement S1: R code and demographic data, Supplement S2: Climatic data.

## Abbreviations

The following abbreviations are used in this manuscript:

MR	Mark-recapture
MSMR	Multi-state mark-recapture
SSM	State space model
HMM	Hidden Markov model
MCMC	Markov chain Monte Carlo
AIC	Akaike’s information criteria
DIC	Deviance information criteria
psrf	Potential scale reduction factor
BFL	Brackenridge Field Laboratory

## Appendix A. Study Species and Field Methods

I mapped out all suitable habitat at BFL, defined as oak-cedar woodland on limestone outcrop, and overlaid it with a grid composed of 25-m × 25-m cells. Of 16 randomly- selected grid points, 14 (22% of 71 total grid points) identified sites containing *C. fruticulosus* that were used for this study. I tagged each *C. fruticulosus* shrub within a contiguous patch at each site and searched each shrub for *A. aidea* caterpillars at each of nine survey dates. For each caterpillar found, I marked the leaf where it was perched with a letter unique within an individual plant, identifying the individual caterpillar. I marked leaves directly on the upper leaf surface with a permanent marker. All host plants in each patch were tagged and searched, so caterpillars that left in search of food most likely perished rather than crawling long distances to neighboring patches.

The particular behavior of *A. aidea* caterpillars provides an opportunity for MR studies (see [27]). Strong host fidelity and investment in constructed perches and shelters by these caterpillars facilitated re-sightings of caterpillars in marked locations on tagged host plants. Similar to other *Anaea* and closely related genera, e.g., *Memphis* and *Consul*, early instars web their feces, called frass, together into a chain that extends from the leaf tip [67,68,69,70]. This frass chain perch dangles from the leaf tip, and is hypothesized to reduce predation risk via camouflage and/or predator-free space [71]. Caterpillars consume the leaf blade, leaving the midrib, to which the frass chain remains attached at the tip. Once the majority of the leaf has been consumed, the caterpillar relocates to an adjacent leaf, constructing a new frass chain as a first order of business. Caterpillars abandon frass chains as they develop into later and larger instars and construct leaf shelters by either webbing leaves together or rolling single leaves [70]. They then feed on adjacent leaves, typically on the same branch as their rolled leaf. When not feeding, caterpillars position themselves snugly in their rolled leaf, protecting their soft bodies from predators, leaving only their hard head capsules facing out (personal observation [47]). Capitalizing on this behavior, I marked each caterpillar-inhabited leaf with a permanent marker and relocated each mark and its caterpillar during each survey.

There were few issues of uncertain identification of caterpillars. Great care was taken in marking the region of plants occupied by a caterpillar, and adjacent leaves were marked when I believed a caterpillar would consume the leaf on which it was perched and move. Where leaves were available, caterpillars reliably relocated to adjacent leaves rather than traversing the shrub. A majority of shrubs hosted only one or two caterpillars, while larger shrubs (ca. 2-m tall) hosted several. In fact, one large shrub hosted 18 caterpillars, but it is important to point out that only a fraction of these caterpillars were present at any point in time. When caterpillars were dense, and identification approached confusion, I could typically distinguish between individuals clearly based on differences in their stage. For example, first cannot become fourth instars in three days and fourth to first instar transitions are impossible. In the few instances where I could not discern the identity of an individual, I treated it as a new individual.

Egg and pupa stages were essentially hidden stages, because they were much less apparent than caterpillar stages. I recorded these stages when I observed them opportunistically, but the demographic rates for these stages in the HMM were largely supported by informative priors estimated from laboratory data. The egg stage appears to have a nonzero observation probability (Figure 3), but this estimate reflects the re-sighting probability of eggs in known locations, not the probability of finding new eggs. Interestingly, *A. aidea* pupates on its host much more frequently during the fall brood than it does in the spring (personal observation[47]). In the spring, fifth instar caterpillars almost exclusively leave their host plant in search of pupation sites in surrounding woodland.

As a measure of size, I measured the length of each caterpillar with a dial caliper, and the lengths of all caterpillars on an individual plant were summed to represent size-scaled density as a potential factor affecting caterpillar survival. Caterpillar length should scale with biomass but this association includes a fair amount of error, because caterpillars can shorten or lengthen their bodies. Density on a single host plant is the relevant metric of density for this system because caterpillars generally stay on a single plant throughout their development and large caterpillars consume more resources than small ones.

## Appendix B. Description of the Demographic and Observation Matrices

The demographic matrix, $P_{X}$, describes the underlying state process of interest, and is a block matrix consisting of matrices J, M, Z, and U. I used elements of the life cycle graph for the juvenile portion of the life cycle in the current model, and arranged only these probabilities for the juvenile stages (egg, first to fifth instar, pupa) in a matrix (J);

(A1) $$J=\left[\begin{array}{ccccccc} \phi_{0}\psi_{00} & 0 & 0 & 0 & 0 & 0 & 0\\ \phi_{0}\psi_{10} & \phi_{1}\psi_{11} & 0 & 0 & 0 & 0 & 0\\ \phi_{0}\psi_{20} & \phi_{1}\psi_{21} & \phi_{2}\psi_{22} & 0 & 0 & 0 & 0\\ 0 & \phi_{1}\psi_{31} & \phi_{2}\psi_{32} & \phi_{3}\psi_{33} & 0 & 0 & 0\\ 0 & 0 & \phi_{2}\psi_{42} & \phi_{3}\psi_{43} & \phi_{4}\psi_{44} & 0 & 0\\ 0 & 0 & 0 & \phi_{3}\psi_{53} & \phi_{4}\psi_{54} & \phi_{5}\psi_{55} & 0\\ 0 & 0 & 0 & 0 & 0 & \phi_{5}\psi_{65} & \phi_{6} \end{array}\right]$$

each column represents a particular stage at time *t*, each row represents the stage at time t+1, and each element $j_{ji}$ represents the probability of survival and growth from stage *i* to stage *j*. Matrix J only represents transitions among living juvenile stages, but I also observed a large number of dead caterpillars.

Matrix M describes transitions from the live state to the dead state, and utilizes the same stage-specific survival and growth probabilities. Each element is a product of the probability of dying, $\mu_{i}=1-\phi_{i}$, and the probability of stage transitioning, $\psi_{ji}$, independent of the probability of dying.

(A2) $$M=\left[\begin{array}{ccccccc} \mu_{0}\psi_{10} & \mu_{1}\psi_{11} & 0 & 0 & 0 & 0 & 0\\ \mu_{0}\psi_{20} & \mu_{1}\psi_{21} & \mu_{2}\psi_{22} & 0 & 0 & 0 & 0\\ 0 & \mu_{1}\psi_{31} & \mu_{2}\psi_{32} & \mu_{3}\psi_{33} & 0 & 0 & 0\\ 0 & 0 & \mu_{2}\psi_{42} & \mu_{3}\psi_{43} & \mu_{4}\psi_{44} & 0 & 0\\ 0 & 0 & 0 & \mu_{3}\psi_{53} & \mu_{4}\psi_{54} & \mu_{5} & 0\\ \mu_{0}\psi_{00} & 0 & 0 & 0 & 0 & 0 & \mu_{6} \end{array}\right]$$

The probability of being dead and in stage *j* at time t+1 depends on stage *i* at time *t*, even though in very few instances caterpillars were able to transition two stages between *t* and t+1. This specification ignores stage-specific survival of the intermediate stage, but including this would upset the implicit time component of survival parameters. These survival parameters represent the probability of surviving three days given initial stage, which is not the same as the probability of surviving a particular developmental stage. Matrix M represents all possible transitions from live stages to dead stages, and ensures that all columns in $P_{X}$ sum to one. One benefit to this parameterization is that we gain information about development by classifying dead stages. The developmental stage of dead caterpillars indicates growth that occurred between the last observation and death. Here the probability of growing from a fifth instar to a pupa then dying is equal to zero (element $m_{66}$).

For both matrices, M and J, the probability of growing from a pupa to an adult was set to zero. Pupae were rarely observed in the field and I was unable to collect data on pupa survival and the pupa to adult transition. The last row in matrix M represents individuals leaving the study, which is the absorbing state. In matrix M, individuals enter the absorbing state by dying in a stage that left no visible remains, i.e., neither eggs nor pupae were observed in the dead state during this study.

The remaining two matrices within $P_{X}$, Z and U, serve to enforce the absorbing state. Matrix Z is has dimensions 7 × 6 (row × column) with all elements set to zero, which represents the impossibility of transitions from the dead state to the live state. Matrix U has dimensions 6 × 6 and represents stage transitions among dead stages, which are similarly impossible. All elements of U are set to zero except for the last row, which represents individuals in the dead state transitioning to the absorbing state. This model classifies dead individuals by developmental stage, and the newly dead state itself is not absorbing. When an individual dies, it moves to the newly dead state for one time step so that it may be observed dead. In the next time step, newly dead individuals move to the absorbing state, dead, indicating it has left the study. Individuals also entered the absorbing state by dying as either an egg or pupa, for which no visible remains were found.

The observation matrix, $P_{O}$, describes the process by which we observe individuals.

(A3) $$P_{O}=\left[\begin{array}{ccccccc} 1-p_{0} & 1-p_{1} & 1-p_{2} & \cdots & 1-p_{10} & 1-p_{11} & 1\\ p_{0} & 0 & 0 & \cdots & 0 & 0 & 0\\ 0 & p_{1} & 0 & \cdots & 0 & 0 & 0\\ \vdots & \vdots & \vdots & \ddots & \vdots & \vdots & \vdots \\ 0 & 0 & 0 & \cdots & 0 & 0 & 0\\ 0 & 0 & 0 & \cdots & p_{10} & 0 & 0\\ 0 & 0 & 0 & \cdots & 0 & p_{11} & 0 \end{array}\right]$$

The columns of $P_{O}$ correspond to the 13 state × stage categories (live (Egg, 1st, 2nd, 3rd, 4th, 5th, Pupa), newly dead (1st, 2nd, 3rd, 4th, 5th), dead) at time t. The rows of $P_{O}$ represent the possible states and stages an individual can be observed as at time *t* (not observed, live (Egg, 1st, 2nd, 3rd, 4th, 5th, Pupa), newly dead (1st, 2nd, 3rd, 4th, 5th)). The sub-diagonal of $P_{O}$ contains the probabilities of being observed in stage *i* ($p_{i}$), given an individual is in stage *i*. The probability of not being observed is represented in the top row and is the complement of each state- and stage-specific probability of being observed, i.e., $1-p_{i}$. Stage identification error was not included, so all other elements of $P_{O}$ are equal to zero, because they represent being observed in a stage *i* when the individual is in fact not in stage *i*.

## Appendix C. Logit Transforms of Survival and Growth Rates

I modeled the covariates for survival in each stage using a logit link, such that the log odds of surviving was equal to a linear function of each covariate.

(A4) $$logit\left(\phi_{i}\right)=b_{1i}+b_{xi}x$$

In this function, *x* was either survey date, which represents the progression of time, or caterpillar density. Reducing this model by setting $b_{xi}$ to zero I obtained the constant survival model with a single parameter for each stage, $b_{1i}$. For parsimony, I did not test non-linear models for either survival or transition probabilities, but this could be a fruitful direction for future research.

Growth probabilities were modeled using a multinomial logit to allow for transitions to multiple stages while requiring that the sum of all possible transitions from *i* to any stage *j* sum to one.

(A5) $$\begin{array}{cc} \psi_{ii} & ={\left[1+exp\left(\alpha_{1i}\right)+exp\left(\alpha_{2i}\right)\right]}^{-1}\\ \psi_{i+1i} & =exp\left(\alpha_{1i}\right){\left[1+exp\left(\alpha_{1i}\right)+exp\left(\alpha_{2i}\right)\right]}^{-1}\\ \psi_{i+2i} & =exp\left(\alpha_{2i}\right){\left[1+exp\left(\alpha_{1i}\right)+exp\left(\alpha_{2i}\right)\right]}^{-1} \end{array}$$

The parameters $\alpha_{1i}$ and $\alpha_{2i}$ for each stage *i* are equal to the log odds of growing one or two stages, respectively, with respect to remaining in stage *i*. These two parameters estimate the three transition probabilities (growth one stage, growth two stages, and stasis), given the restriction that the probabilities sum to one ($\psi_{ii}=1-\psi_{i+1i}-\psi_{i+2i}$). To test for environmental effects, I formulated each parameter $\alpha_{1i}$ and $\alpha_{2i}$ as a linear function of *x*,

(A6) $$\begin{array}{cc} \alpha_{1i} & =a_{1i}+a_{xi}x\\ \alpha_{2i} & =a_{2i}+a_{xi}x \end{array}$$

where *x* was either a temporal effect (date) or air temperature, and $a_{xi}$ describes the slope of the relationship between α and *x*, which is equal for both $\alpha_{1i}$ and $\alpha_{2i}$. Setting $a_{xi}$ to zero reduces this model so that growth probabilities are constant. For certain transitions (fourth to fifth and fifth to pupa), the probability of growing two stages is zero, so the binomial logit was used for these probabilities with only two possible outcomes.

I was able to fit these models for the most apparent stages, first to fifth instars, so I specified uninformative prior distributions for these stages (*i* in 2 to 6). Here, I specified normal distributions, Normal(0,100), for all linear parameters, $b_{1i}$, $b_{xi}$, $a_{1i}$, $a_{2i}$, $a_{xi}$, and uniform distributions, Uniform(0,1), for observation parameters, $p_{i}$. Given significantly fewer observations of egg and pupae, I restricted the range of possible observation probabilities for egg and pupa, Uniform(0.02,0.4), and I did not fit linear models for survival of these stages, $\phi_{1}$ and $\phi_{7}$, specifying instead a uniform distribution between zero and one for each. Transitions from egg to first instar, egg to second instar, and fifth instar to pupa were rarely observed in the field, so I estimated these parameters by fitting a HMM to observations of 150 caterpillars raised in the laboratory. Laboratory conditions were set to mirror the mean diurnal temperature and daylength cycles that were observed in the field. With these estimates I specified the following informative distributions.

(A7) $$\begin{array}{cc} a_{11} & ∼Normal(1.0,1)\\ a_{21} & ∼Normal(-1.9,1)\\ a_{76} & ∼Normal(-0.93,1) \end{array}$$

## Appendix D. Per Capita Predation Rate

To better understand how predation rates changed over time at my sites, I collated the number of caterpillar corpses, $n_{dead}$, and living caterpillars, $n_{live}$, observed at each census. I then estimated the per capita predation rate, the ratio of dead to live caterpillars, as a log-linear model of time.

(A8) $$\begin{array}{cc} n_{dead} & ∼Poisson(\lambda )\\ log(\lambda ) & =c_{1}+c_{2}t+log\left(n_{live}\right) \end{array}$$

I used similar MCMC methods as described above, and checked all diagnostics to ensure convergence. Parameter values (and credible intervals) were $c_{1}=-4.43\left(-4.96,-3.96\right)$ and $c_{2}=0.112\left(0.083,0.143\right)$.
