# Supplementary material for: Estimating Density and Temperature Dependence of Juvenile Vital Rates Using a Hidden Markov Model
Source: Insects. 2017 May 15;8(2):51. doi: 10.3390/insects8020051 (PMC5492065; doi:10.3390/insects8020051)
Supplement: Supplementary file 1 [file insects-08-00051-s001.zip › McElderry_MR_caterpillars_Insects_Supplements_2017.pdf]

Table S1 Model selection table showing the information criteria (AICc and ΔAICc), and relative weighting (Weight) for each model.

**Survival**	**Transition**	**# Par**	**AICc**	**ΔAICc**	**Weight**
ϕ(st(E,L),date)	ψ(stage)	28	1588.86	0	0.5
ϕ(stage)	ψ(stage)	29	1588.87	0.01	0.5
ϕ(st(E,L))	ψ(stage)	26	1603.58	14.72	0
ϕ(stage,density)	ψ(stage,temp)	38	1605	16.14	0
ϕ(st(E,L),density)	ψ(stage,temp)	32	1606.37	17.51	0
ϕ(st(E,L))	ψ(stage,temp)	30	1607.96	19.1	0
ϕ(stage)	ψ(stage,date)	33	1608.09	19.23	0
ϕ(st(E,L),date)	ψ(stage,temp)	32	1612.62	23.76	0
ϕ(st(E,L),density)	ψ(stage,date)	32	1617.25	28.39	0
ϕ(stage,date)	ψ(stage,date)	38	1620.53	31.67	0
ϕ(stage)	ψ(stage,temp)	33	1621.13	32.27	0
ϕ(stage,density)	ψ(stage,date)	34	1629.24	40.38	0
ϕ(st(E,L),date)	ψ(stage,date)	32	1630.5	41.64	0
ϕ(st(E,L),density)	ψ(stage)	28	1633	44.14	0
ϕ(stage,date)	ψ(stage,temp)	38	1641.69	52.83	0
ϕ(stage,density)	ψ(stage)	38	1647.73	58.87	0
ϕ(st(E,L))	ψ(stage,date)	30	1663.16	74.3	0
ϕ(stage,date)	ψ(stage)	34	1665.02	76.16	0

Note: Models were manipulated such that either survival (ϕ) or transition (ψ) probabilities were functions of stage, survey date, density, or temperature. For survival only, stage was grouped into early and late instar, represented as, st(E,L). All models used stage-dependent probability of observation.

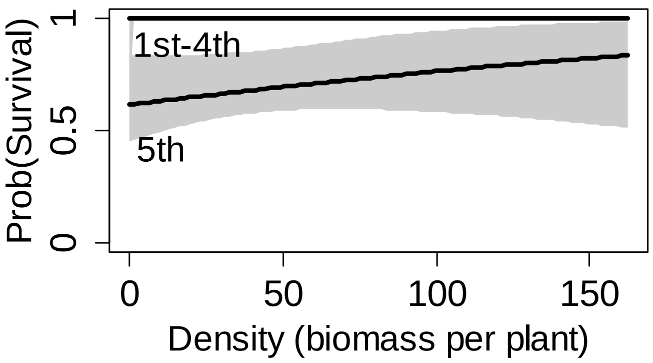

Figure S1 Change in mean survival of *A. aidea* in response to size-scaled density. Size-scaled density was estimated by the sum of the lengths of all caterpillars on a plant at each survey.

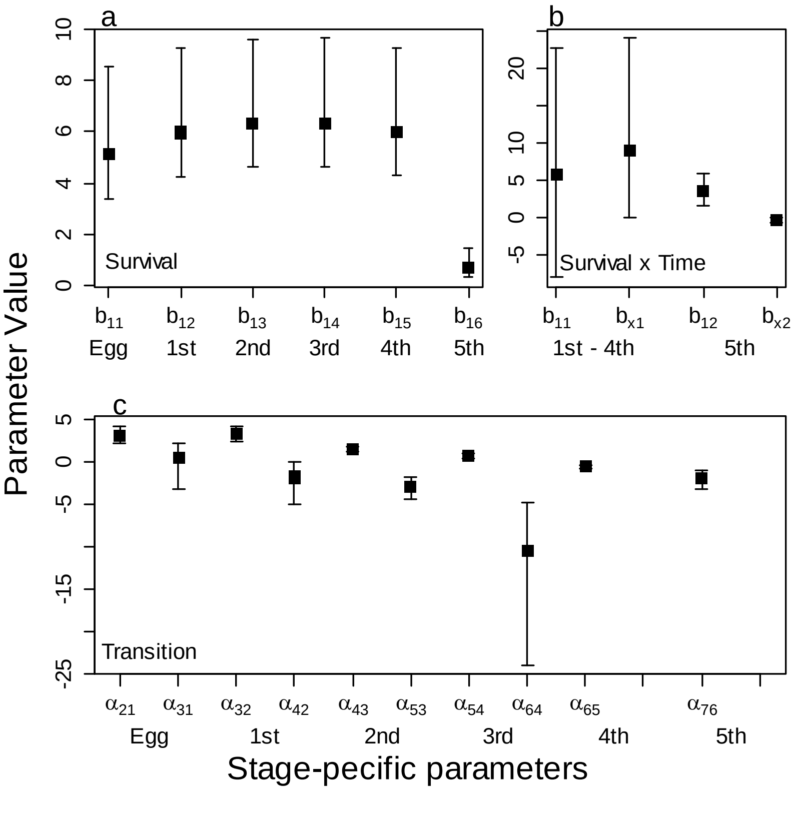

Figure S2 Untransformed parameter estimates for the two highest ranking models. Parameters in (**a**) and (**c**) are for model ϕ(stage) ψ(stage), parameters in (**b**) are for model ϕ(st(E,L),date) ψ(stage). Parameter label and indexing follow equations in Appendix C.
